# A requirement for astrocyte IP_3_R2 signaling for whisker experience-dependent depression and homeostatic upregulation in the mouse barrel cortex

**DOI:** 10.3389/fncel.2022.905285

**Published:** 2022-08-25

**Authors:** John B. Butcher, Robert E. Sims, Neville M. Ngum, Amjad H. Bazzari, Stuart I. Jenkins, Marianne King, Eric J. Hill, David A. Nagel, Kevin Fox, H. Rheinallt Parri, Stanislaw Glazewski

**Affiliations:** ^1^School of Life Sciences, Keele University, Keele, United Kingdom; ^2^College of Health and Life Sciences, Aston University, Birmingham, United Kingdom; ^3^Neural Tissue Engineering Group, Institute for Science and Technology in Medicine (ISTM), Keele University, Keele, United Kingdom; ^4^Aston Medical School, Aston Medical Research Institute, Aston University, Birmingham, United Kingdom; ^5^School of Biosciences, Cardiff University, Cardiff, United Kingdom

**Keywords:** Hebbian plasticity, homeostatic plasticity, synaptic plasticity, LTD (long term depression), LTP (long term potentiation), BCM, somatosensory

## Abstract

Changes to sensory experience result in plasticity of synapses in the cortex. This experience-dependent plasticity (EDP) is a fundamental property of the brain. Yet, while much is known about neuronal roles in EDP, very little is known about the role of astrocytes. To address this issue, we used the well-described mouse whiskers-to-barrel cortex system, which expresses a number of forms of EDP. We found that all-whisker deprivation induced characteristic experience-dependent Hebbian depression (EDHD) followed by homeostatic upregulation in L2/3 barrel cortex of wild type mice. However, these changes were not seen in mutant animals (IP_3_R2^–/–^) that lack the astrocyte-expressed IP_3_ receptor subtype. A separate paradigm, the single-whisker experience, induced potentiation of whisker-induced response in both wild-type (WT) mice and IP_3_R2^–/–^ mice. Recordings in *ex vivo* barrel cortex slices reflected the *in vivo* results so that long-term depression (LTD) could not be elicited in slices from IP_3_R2^–/–^ mice, but long-term potentiation (LTP) could. Interestingly, 1 Hz stimulation inducing LTD in WT paradoxically resulted in NMDAR-dependent LTP in slices from IP_3_R2^–/–^ animals. The LTD to LTP switch was mimicked by acute buffering astrocytic [Ca^2+^]_*i*_ in WT slices. Both WT LTD and IP_3_R2^–/–^ 1 Hz LTP were mediated by non-ionotropic NMDAR signaling, but only WT LTD was P38 MAPK dependent, indicating an underlying mechanistic switch. These results demonstrate a critical role for astrocytic [Ca^2+^]_*i*_ in several EDP mechanisms in neocortex.

## Introduction

**FIGURE 1:**
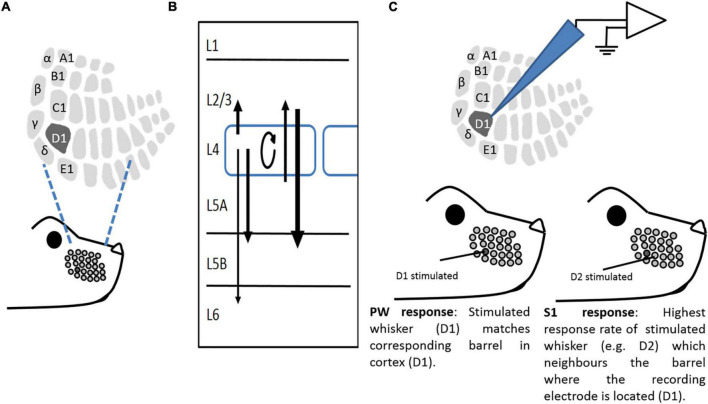
A study model. **(A)** Representation of a whisker pattern on mouse snout, illustrating corresponding somatotopic representation in L4 barrel cortex. **(B)** Schematic of major vertical connections within a barrel in the barrel cortex. Arrows show connectivity between layers, with their strength expressed as line thickness. **(C)** An illustrative example of *in vivo* recording paradigms. Extracellular recordings were made from neurons of the barrel cortex. The “Principal” whisker (PW) is the whisker that primarily projects to the recorded barrel (in this case, D1). A “surrounding” whisker (SW) is a whisker that is adjacent to the Principal whisker (in this case, D2).

Mice explore the environment using their whiskers. Signals in sensory afferents triggered by whisker deflections are ultimately transmitted to somatotopically organized populations of cells in the primary somatosensory cortex known as the barrel cortex (Figure 1 modified from; Fox, 2002). Alterations in whisker experience can induce plastic changes in neurons of the barrel cortex—experience-dependent plasticity (EDP). EDP is a fundamental feature of sensory neocortex (e.g., somatosensory, auditory, and visual), indicating that cortical circuits and synapses are endowed with the capacity to change in response to sensory input changes from the environment. Adolescent mice (1–2 months old) exhibit two general forms of EDP in layers 2/3 of barrel cortex: Hebbian plasticity and homeostatic plasticity (HP) (Glazewski and Fox, 1996; Sims et al., 2015; Glazewski et al., 2017). The former refers to changes in neuronal transmission at individual synapses, which are thought to sculpt neuronal networks during development and enable information coding and storage (Fox, 2008; Espinosa and Stryker, 2012). This type of plasticity has two forms: Experience-dependent Hebbian potentiation (EDHP) and experience-dependent Hebbian depression (EDHD), which closely resemble long-term potentiation (LTP) and LTD expressed *in vitro* (Glazewski and Fox, 1996; Hardingham et al., 2003, 2008). In contrast, HP is most often a global phenomenon, being manifested at a cell-wide or cell-population level rather than at individual synapses (Turrigiano et al., 1998; Turrigiano, 2012; Glazewski et al., 2017). HP is posited to provide a negative feedback mechanism to maintain the activity of neuron/neuronal network within a set operating range.

Current explanatory models for the mechanisms of EDP are still predominantly neuro-centric. However, in recent years, and not without controversy (Fiacco and McCarthy, 2018; Savtchouk and Volterra, 2018), increasing evidence mostly from slice studies has emerged regarding the role of astrocytes in synaptic communication and several forms of Hebbian plasticity (Henneberger et al., 2010; Takata et al., 2011; Chen et al., 2012; Min and Nevian, 2012; Araque et al., 2014; Perea et al., 2014; Pankratov and Lalo, 2015; Navarrete et al., 2019). However, very little is still known about the potential roles of astrocytes in different forms of EDP, particularly from *in vivo* studies.

Anatomically, astrocytes are ideally placed for such roles, with numerous, heavily ramified and often ultrathin astrocytic processes ensheathing a large proportion of synaptic spines in various areas of the brain (Bushong et al., 2002), including the neocortex (Bernardinelli et al., 2014; Papouin et al., 2017).

Astrocytes express both ionotropic (Lalo et al., 2011) and metabotropic receptors (Volterra et al., 2014; Bazargani and Attwell, 2016; Foley et al., 2017) that sense synaptic input resulting in [Ca^2+^]_*i*_ elevations. A major pathway in eliciting these is the Gq-PLC-IP_3_ pathway (Gq-Phospholipase C - inositol triphosphate receptor), which can consequently lead to the release of gliotransmitters affecting neuronal firing.

In astrocytes, somatic [Ca^2+^]_*i*_ elevations are predominantly mediated by the IP_3_R2 subtype (Srinivasan et al., 2015); however, there is a lack of expression of this subtype in neurons (Sharp et al., 1999; Holtzclaw et al., 2002; Petravicz et al., 2008; Zhang et al., 2014). In this study, in the barrel cortex model system, we used pharmacological, opto-, chemogenetic, and KO approaches with electrophysiological recording *in vivo* and in slice preparations, together with whisker deprivation paradigms that are known to trigger Hebbian and HP.

We found that opto- and chemo-genetic activation of astrocyte Gq-coupled signaling pathways increases neuronal activity *in vivo* and *in vitro*, and that loss of IP_3_R2 leads to the impairment of EDHD and LTD as well as suppression of experience-dependent homeostatic upregulation. This demonstrates a critical role for astrocytes in neocortical plasticity.

## Methods

All experiments were approved by local ethical review and performed in accordance with the United Kingdom Animals Scientific Procedures Act of 1986 and current EU legislation. The experiments were planned with consideration for the 3 Rs, replacement, and refinement and reduction. All animals used in the experiments were male and female mice of the Bl6J background. IP_3_R2^–/–^ mice were imported from the laboratory of Prof. Araque (Cajal Institute, Madrid), with the permission of their creator, Prof. Ju Chen (San Diego), re-derived into the C57Bl6/6J background and amplified by crossing homozygotes. Genotyping during the project was conducted by Transnetyx (TN, United States).

### *In vivo* experiments

#### Extracellular recording *in vivo*

Single-whisker experience (SWE), achieved by removing all but one whisker (D1 in this study), has been shown to induce EDHD and EDHP (Glazewski et al., 1996). All-whisker deprivations in WT animals are known to induce immediate EDHD followed by homeostatic upregulation (Glazewski et al., 2017). Some data from all-whisker deprived for up to 14 days and undeprived WT animals have been previously published (Glazewski et al., 2017). The number of animals used for all of the *in vivo* experiments, including the number of cells recorded from Layers 2/3, is detailed in Table 1 below.

**TABLE 1:** Number of animals and cells recorded from (in brackets) for each of the *in vivo* experiments.

Experiment	Number of days of whisker deprivation	Number of animals and layer 2/3 cells (in brackets) recorded, for each of the *in vivo* experiments		
		WT	IP_3_R2^–/–^	GluA1^–/–^
Control	N/A	9 (122)	8 (105)	6 (45)
SWE	18 (plus 5–9 days regrowth)	11 (132)	9 (130)	N/A
All-whisker deprivation	1	6 (97)	6 (105)	4 (58)
All-whisker deprivation	3	5 (57)	7 (110)	1 (15)
All-whisker deprivation	7	3 (43)	6 (99)	4 (55)
All-whisker deprivation	14	7 (49)	6 (105)	N/A
All-whisker deprivation	25	3 (58)	5 (80)	N/A
All- whisker deprivation	32	3 (56)	5 (90)	N/A
Non-injected optogenetic activation of astrocytes	N/A	3 (13)	N/A	N/A
Melanopsin optogenetic activation of astrocytes	N/A	11 (32)	N/A	N/A
DREADD activation of astrocytes	N/A	Control: 3 (3*) DREADD: 2 (2*)	N/A	N/A

In parallel recordings from Layer 4 of IP_3_R2^–/–^ animals, 148 cells were recorded across all experimental groups, i.e., undeprived controls and 1, 3, 7, 14, 25, and 32 days, all-whisker deprived. *Evoked local field potentials were recorded for the DREADD experiments, as such the number of cells is not known; therefore, the number of recording sites is shown instead.

#### Whisker deprivation

Two deprivation paradigms were used in this study, and each had its own variation of the deprivation method. SWE and all-whisker deprivations were performed unilaterally on mice aged 28–30 days under brief isoflurane (Abbott Ltd.) anesthesia. To evoke Hebbian potentiation and depression, all-whiskers except D1 were removed by carefully applying steady tension to their bases without compromising the integrity of innervation (Figure 1; Li et al., 1995). The whiskers were inspected daily for regrowth and re-deprived if necessary. After 18 days of deprivation, the whiskers were allowed to regrow for 5–9 days before recording. To evoke EDHD, followed by homeostatic rebound, all the whiskers were trimmed flush with the skin using surgical micro-scissors, and re-trimmed daily to the same length for 1, 3, 7, 13–15, 25, and 32 days. On the day of recording, the original cut whisker was re-attached to the stub with use of cyanoacrylate glue (Henkel Ltd., United Kingdom), and deflected during extracellular recording.

#### Anesthesia and surgery

For all experiments, anesthesia was induced with isoflurane and maintained with urethane (Sigma-Aldrich) (1.5 g/kg of body weight), complemented with trace amounts of acepromazine (Novartis, Switzerland, injected i.p.) (Glazewski et al., 1996). All the recordings were performed at Stage III-3 of anesthetic depth, characterized by sluggish hind limb pinch withdrawal reflex and corneal blink reflex present (Fox and Armstrong-James, 1986). If necessary, 10% of the initial dose of urethane/acepromazine mixture was administered to maintain required anesthetic depth. Body temperature was maintained at 37°C throughout the experiment using a homeothermic blanket system (Harvard Apparatus). Before recording, an anesthetized animal was secured in a stereotaxic frame (Narishige Ltd.), the scalp was retracted, and the skull carefully thinned over the barrel cortex with a dental drill (0–3 mm caudal from the bregma and 1.5.–3 mm lateral from the midline). Before each electrode penetration, a small hole was made in the thinned skull using the tip of a small hypodermic needle. The skull and the recording area were kept moist during the recording session with phosphate buffered saline.

#### Electrodes, stimulation, and recording

Custom-made glass-insulated and chromic acid-sharpened carbon fiber microelectrodes were used to record extracellular potentials from the cortex (Armstrong-James et al., 1980). Electrodes were lowered perpendicular to the cortical surface and single unit activity sampled across layers 2–4 (0–450 μm down the pial surface) at roughly 50-μm-depth intervals. Action potentials were amplified (2,000×) and filtered (0.7–7 KHz), with a 50-Hz notch filter using a Neurolog system (Digitimer). In the case of Designer Receptor Exclusively Activated by Designer Drug (DREADD) experiments, local field potentials (LFPs) were recorded with 0.1-Hz high pass filter along with a Humbug 50-Hz noise eliminator (Digitimer). Stimulation consisted of a 1° vertical, upward deflection of a whisker (i.e., 200-μm whisker deflection at 10 mm from the base), lasting 10 ms. This stimulation intensity evokes a half-maximal response of principal whiskers (PW—i.e., whiskers represented in recorded columns in contrast to the surround whiskers (SW) (Figure 1C; Armstrong-James and Fox, 1987). This standard deflection was calibrated and monitored throughout all the experiments using a laser optical displacement sensor (Micro-Epsilon Ltd., United Kingdom). To account for a low fidelity of cortical responses, 50 stimuli at 1 Hz were applied per whisker stimulated. The stimuli were delivered using a fast, piezoelectric bimorph wafer attached to a lightweight glass capillary driven from a voltage source (DS-2, Digitimer, United Kingdom) and controlled by Spike2 software (CED). Both spontaneous and whisker-evoked firing was used to isolate a given cell with the use of a window discriminator.

#### Opto-/chemogenetics

A custom-designed optogenetic Adeno-associated virus (AAV)(5) vector expressing mouse melanopsin isoform 1 (OPN4, RefSeq accession BC139827) under the control of the Glial fibrillary acidic protein (GFAP) 0.7 promoter was made for us by Vector Biolabs (Malvern, PA, United States). For visualization of the transduced cells, Cyan fluorescent protein (CFP) was linked to the OPN4 using a T2A self-cleaving peptide sequence in the viral construct. The DREADD was AAV(5)-GFAP-HA-hM3D(Gq)-IRES-mCitrine (Dr. Bryan Roth lab) and was supplied by DUKE University viral vector core (NC, United States). AAVs were stereotactically injected into the barrel cortex of 3–4-week-old mice using a beveled glass capillary connected to Nanoject II auto-nanoliter injector (Drummond Sci., United States). The mice were anaesthetized using isoflurane and secured in a stereotaxic frame. The scalp was carefully retracted, and the skull thinned with use of a dental drill (0–3 mm caudal from the bregma and 1.5–3 mm lateral from the midline). Two injection sites centered over the D1 barrel column were made along its vertical axis (300, 200, and 100 μm down from the pial surface) with 500 nl each injected over 3–4 min. The localization of transgenes in the barrel cortex was tested by apposing cytochrome oxidase and transgene reporter images using computer-assisted Axio Zoom V16 with an AxioCam ICm1 camera and the ZEN software (Blue Ed., v.1.1.1.0) (Carl Zeiss GmbH) after mounting DAPI counter-stained sections with use of a Vectashield antifade mounting medium (Vector labs). The melanopsin transgene was activated using the Spike2-controlled PlexBright optogenetic stimulator system (Plexon Inc.). During the 30 min light onstage of recording, 470 nm light stimuli (13 mW-measured power at a fiber tip) were delivered at 5 Hz (a 150 ms-long pulse with a 50 ms-long gap) for the first 30 s of each minute through the thinned skull, while action potentials were recorded from L2/3 of the barrel cortex. The same protocol was used in non-injected mice as a control.

The DREADD was activated with clozapine N-oxide (CNO) i.p injection of 200 μl of 0.15 mg/ml per 30 g of mouse weight, ∼1 mg/kg, dissolved in sterile saline (Amersham, United Kingdom), while LFPs were recorded from the L2/3 barrel cortex before and after CNO injection in response to the standard principal whisker deflections defined above. The same protocol was followed for CNO injection in the control mice.

#### Post-recording immunohistochemistry

At the end of each electrode penetration, a small lesion (1 μA, direct current (DC), 10 s, tip negative) was made 300 μm below the pial surface to mark the location of each electrode penetration. After each experiment, the animals were deeply anaesthetized with urethane and perfused through the heart with small amounts of 0.1-M phosphate-buffered saline followed by 50 ml of 4% buffered solution of paraformaldehyde. The brain was carefully removed, the cortex peeled off and flattened as described previously (Strominger and Woolsey, 1987) and left overnight in buffered solution of 4% paraformaldehyde containing 30% sucrose at 4°C. In the morning, this solution was replaced with buffered solution of 30% sucrose and stored for up to 2 weeks at 4°C. Sections of 40-μm thickness were cut tangentially to the surface of the flattened cortex using a freezing microtome, and the tissue was reacted for cytochrome oxidase (Wong-Riley, 1979). The stained sections were analyzed for lesion location and *post-hoc* correction of recording depth using the barrel pattern as reference. Only the cells located in the barrel columns and up to and including 300 μm below the pial surface were classified as Layer 2/3 and included in analysis.

#### Opto/chemogenetic immunohistochemistry

To check the location of the recording electrode relative to transgene expression for the optogenetic and chemogenetic experiments, a small lesion was made as described above. Slices (40 μm) thickness was incubated in blocking solution [Phosphate buffered saline (PBS), 5% normal donkey serum, 0.3% triton X-100; 2 h; room temperature (RT)], followed by incubation with a primary antibody in blocking solution (rabbit anti-GFAP, 1:100, Z0334, Dako, United Kingdom). The sections were washed three times in PBS (20 min per wash), incubated with blocking solution (2 h; RT) and then incubated with a secondary antibody (a CF350-labeled donkey anti-mouse IgG secondary antibody, 1:200, 450-nm emission, SAB4600009, Sigma-Aldrich, United Kingdom). Lastly, the sections were washed three times with PBS (20 min per wash), transferred to slides, mounted with Dibutylphthalate polystyrene xylene (DPX) (Sigma-Aldrich), and coverslipped. The slices were imaged using computer-assisted Axio Zoom V16 with the AxioCam ICm1 camera and the ZEN software (Blue Ed., v.1.1.1.0) (Carl Zeiss GmbH). The slices then underwent the cytochrome oxidase staining procedure outlined above to confirm the position of the electrode within the virus area. Only recordings that were within the area of virus were included in the data analysis. The extent of virus spread was analyzed using ImageJ. To determine specific astrocyte expression, both transgenes were examined by incubation of tissue sections (40-μm thickness) in blocking solution (PBS, 5% normal donkey serum, 0.3% triton X-100; 2 h; RT) followed by incubation with primary antibodies in blocking solution (rabbit anti-GFAP, 1:100, Z0334, Dako, United Kingdom; mouse anti-NeuN IgG, 1:100, MAB377, clone A60, Millipore, United Kingdom). The sections were then washed three times (PBS, 20 min per wash), incubated with blocking solution (2 h; RT) and then incubated with secondary antibodies (the CF350-labeled donkey anti-mouse IgG secondary antibody, 1:200, 450-nm emission, SAB4600009, Sigma-Aldrich, United Kingdom; the Cy3-conjugated donkey anti-rabbit secondary antibody, 1:200, Jackson Immunoresearch, United States, 24 h 4°C). Lastly, the sections were washed three times with PBS (20 min per wash), transferred to slides, mounted with DPX (Sigma-Aldrich), and coverslipped. The expression of both transgenes was assessed by counting the number of astrocytes and neurons that express the transgene using ImageJ.

#### Data analysis

The magnitude of whisker-evoked responses, expressed in spikes per stimulus (s/s), was calculated using Spike2 software (CED). Action potentials were counted between 3 and 53 ms post-stimulus and corrected for spontaneous activity recorded in a 50-ms time window immediately prior to the stimulus. Responses of Layer 2/3 neurones to whisker stimulation were averaged within each animal and animal averages compared across treatment groups. Responses of Layer 4 neurones, less numerous due to the small span of Layer 4, were averaged inside the group of treatment. The appropriate statistical analysis was applied after testing the data sets for normality and homogeneity (GraphPad Prism, Matlab).

### *In vitro* experiments

For slice experiments, both male and female mice were used, humanely killed by isoflurane overdose, followed by cervical dislocation.

#### Slice preparation and maintaining solutions

Slices of the mouse barrel cortex were prepared as described previously (Pirttimaki et al., 2017). Briefly, after removal from the skull, the brain was glued with cyanoacrylate adhesive to a metal block and submerged in the bath of a Campden 7,000 vibroslicer (Campden Instruments). The bathing solution contained the following (in mM): NaCl 120, NaHCO_3_ 25, KCl 1, KH_2_PO_4_ 1.25, MgSO_4_ 5, CaCl_2_ 2, and glucose 10 and was maintained at <5°C. Cortical slices in the coronal plane (all at 300 μm); all the slices were stored in a 95% O_2_, 5% CO_2_ bubbled solution of identical composition at RT. After a 1-h recovery period, the experiments were performed in an artificial CSF (aCSF) solution containing the following (in mM): NaCl 120, NaHCO_3_ 25, KCl 2, KH_2_PO_4_ 1.25, MgSO_4_ 1, and CaCl_2_ 2. aCSF chemicals were obtained from Sigma-Aldrich, except D-AP5, N-Methyl_D-Aspartate (NMDA), and D-Serine, which were obtained from Ascent Scientific (Bristol, United Kingdom).

#### Fluorescence imaging and optogenetic stimulation

The slices and primary cells were incubated with 10-μM Fluo-4AM (Invitrogen) as previously described (Gould et al., 2014; Pirttimaki et al., 2017). Astrocytes in slice preparations were identified by their preferential loading with Fluo-4, and morphological characteristics. Ability to identify was confirmed following single astrocyte patch pipette filling with solution containing Alexa488. Astrocytes and neurons in primary culture were morphologically characterized using immunocytochemistry (ICC) staining for TUJ1 and S100b. TUJ1-stained cells had small diameter somas (∼10 μm) and 2- to 3-long processes. S100b-positive cells displayed characteristic astrocytes in culture morphology of a large (∼30 μm) diameter with tile-like arrangement. The morphological features were used to identify neurons for analysis of culture experiments. The slices were placed in a recording chamber on a moveable bridge platform (Luigs and Neumann GMBH, Ratingen, Germany) and perfused with aCSF at ∼1 ml/min. Fluorescence imaging was conducted using a Nikon FN1 upright microscope with Light emitting diode (LED)-based illumination (Cairn Research) and acquired using a Hamamatsu ORCA-ER camera. Contrast and brightness were also adjusted to enhance morphological details. In these experiments, acquisition was controlled with HC Image software (Hamamatsu). Images were typically acquired at 0.2 Hz. Imaging experiments were conducted at 32°C. Melanopsin construct stimulation was conducted with LED (450–492 nm) with measured power at the objective of ∼10-mW for 10 s at a time.

#### Slice electrophysiology

All slice recording experiments, patch, and MEA were conducted at 32 ± 1°C. Patch-clamp recordings were made using borosilicate pipettes (Harvard Instruments; 2–4 MΩ) with an internal solution containing the following (in mM): KMeSO_4_ 120, HEPES 10, EGTA 0.1, MgCl_2_ 2, Na_2_ATP 4, and GTP 0.5 with osmolarity adjusted to 295 mOsm with KCl. Currents were recorded using a Multiclamp 700B amplifier, and data were acquired and analyzed using PClamp 9 (Molecular Devices). For synaptic plasticity experiments, extracellular field recordings were made from Layers 2/3; synaptic stimulation was conducted using a Multichannel systems STG 1002 stimulator with a bipolar electrode placed in Layer 4 (Figure 1C). LTP induction was achieved using a standard θ-burst protocol, while LTD was induced using a standard protocol of 900 pulses at 1 Hz, both at half-maximal response. The degree of plasticity was measured at 30 min, following end θ-burst or 1-Hz protocol and compared to field excitatory postsynaptic potential (fEPSP) at the same time point in slices (control) where induction protocols were not applied. Acute inhibition of astrocyte calcium signaling was conducted as previously described (Pirttimaki et al., 2011; Pirttimaki and Parri, 2012), where a single astrocyte was patch-clamped with an electrode containing 20-mM BAPTA (dissolved in standard internal solution: KMeSO_4_ 120, HEPES 10, EGTA 0.1, MgCl_2_ 2, Na_2_ATP 4, and GTP 0.5) and 100-μM Alexa 488 to determine extent of syncytial buffer spread. In the case of the 20-mM BAPTA containing solution, more KOH was required to balance pH compared to standard internal solution. Following pH-ing, the solution was diluted ∼10% with distilled water to bring osmolarity to the appropriate range. Consequently, we estimated that, for BAPTA containing internals, other ion concentrations will be ∼10% lower concentration than indicated by the recipe.

#### Microelectrode array recordings

For MEA recordings, we used a MED64 system (Alpha MED Scientific, Japan). The glass MEA probes consisted of 64 platinum microelectrodes arranged in an 8×8 pattern. The width of each electrode was 50 μm, and the inter-electrode distance was 150 μm.

The probes were coated with 0.1% w/v polyethyleneimine (PEI) in a borate buffer and left overnight (>12 h) at RT before initial use. Somatosensory cortex slices, including the barrel cortex, were placed on the electrodes, and using a “perfusion cap” with an inlet and an outlet, the slices were perfused with aCSF. The MEA was placed on an anti-vibrational table with a Faraday cage to eliminate any source of vibrational or electrical noise. To selectively stimulate L4 and record fEPSPs from L2/3, the slices were positioned on the MEA probes using an inverted microscope (10-× objective lens).

Population activity L2/3-evoked fEPSPs were recorded from eight electrodes across two rows of electrodes from each layer.

A period of 10 min was allowed for the slice to adjust to temperature and recording aCSF conditions. The standard fEPSP measurement parameters of recording were used (the input range: 5 mV, lower-cut frequency: 1 Hz, higher-cut frequency: 10 KHz). Synaptic stimulation was achieved by delivering 0.2-ms biphasic pulses to one electrode on which L4 was situated. The stimulation current was set to 50% of a maximal fEPSP rising slope (a 10–90% slope) determined through an initial input-output (I/O) curve for each slice. The I/O-curve template consisted of 20 traces (trace interval: 20 s, frequency: 0.05 Hz), starting from 10 μA with an incremental increase of 5 μA.

A baseline recording of 20 min was obtained for each slice before any chemical or electrical intervention to provide a slice-specific reference measure for data normalization. A relatively low stimulation frequency (period: 2 min, frequency: 0.008 Hz) was used for both: baseline and response measurements following plasticity induction to avoid potential continuous stimulation-related effects on slice responses. The same low frequency stimulation (LFS) protocols described for glass electrode recordings were implemented, but using 0.2-ms biphasic pulses and the functional stability of induced plasticity was monitored up to 1 h post-induction. A major advantage of MEA recordings is that cells on different recording electrodes represent different populations. Averaging multiple electrodes in a slice reduces variation and so increases the outcome signal to the noise ratio. For plasticity experiments, this means that there is a reduction in the number of experiments required to detect a statistically significant change. This also results in a reduction in the number of animals used in line with 3 Rs considerations. To closer emulate the *in vivo* cell environmental situation, pharmacological blockers of inhibitory GABA-ergic transmission were not used in slice experiments in this study.

#### Data analysis

Voltage and current recordings were analyzed in Clampfit (Molecular Devices). Images were analyzed in HC Image (Hamamatsu). MEA data were acquired and analyzed using the Mobius software (AlphaMED, Japan). Data were analyzed using the fEPSP rising slope. The 10–90% fEPSP slope values were normalized to the 20-min pre-induction baseline mean value for each recorded electrode, which were then averaged (eight electrodes) for each time point/slice. The last 5 time-point values 1 h post-induction of LTD (50–60 min) were averaged per electrode, and the eight electrodes mean values were then averaged per slice. This results in one mean value per slice; this represents the number of independent repeats. Degree of plasticity was measured at 60 min following end θ-burst or 1-Hz protocol and compared to fEPSP at the same time point in slices (control) where induction protocols were not applied. Data traces and values were imported into Sigmaplot 13 (Jandel).

#### Data availability

Source data are provided as a source data file. The datasets generated during and/or analyzed during the current study are available at Aston Data Explorer: researchdata.aston.ac.uk.

## Results

### Opto- and chemogenetic astrocyte activation modulates cortical spike and synaptic activity.

To identify and determine potential roles for astrocytes in EDP, we first asked the question whether direct astrocyte activation could modify barrel cortex neuronal and synaptic activity.

Gq-IP_3_-coupled membrane receptors induce calcium release from intracellular stores and are part of a major pathway by which astrocytes detect synaptic activity (Porter and Mccarthy, 1996). Activation of the Gq-IP_3_ pathway by metabotropic receptors (Volterra et al., 2014; Bazargani and Attwell, 2016) modulates synaptic transmission *via* gliotransmitter release (Perea and Araque, 2007; Pirttimaki and Parri, 2012), and persistent activation of the Gq-IP_3_ system leads to the modulation of gliotransmitter release itself (Pirttimaki et al., 2011). We, therefore, sought to establish whether activation of Gq- IP_3_-coupled pathways in astrocytes was capable of modulating astrocyte excitability and, hence, local neuronal and synaptic activity in the barrel cortex. We used an opto and chemo-genetic approach to achieve this.

**FIGURE 2:**
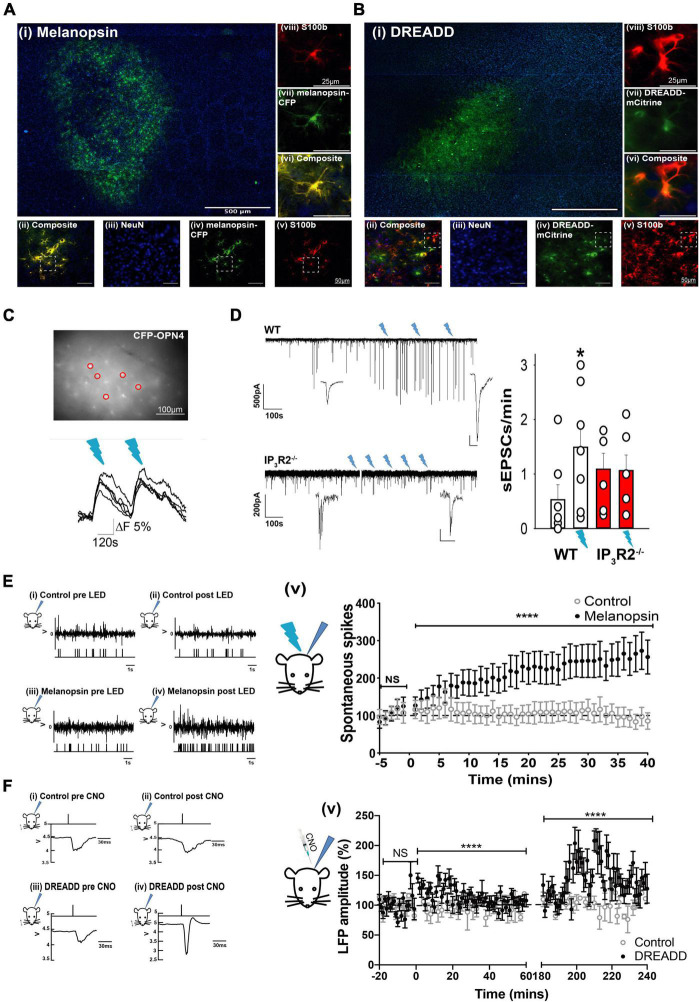
Opto- and Chemogenetically activated astrocytes drive synaptic and spike activity in L2/3 neurons of the barrel cortex *in vitro* and *in vivo*. **(A)** Images showing Melanopsin expression in astrocytes: (i) Low magnification image displaying extent of Melanopsin staining in horizontal slices. (ii) Composite image showing co-expression (yellow) of melanopsin -CFP and S100b. (iii) image of NeuN staining (blue). (iv) Melanopsin-CFP (green). (v) S100b staining (red). Images (vi) are expanded region from (ii). (vii) The expanded region from (iv). (Viii) The expanded region from (v). **(B)** Images showing DREADD expression in astrocytes: (i) Low magnification image displaying extent of DREADD staining in horizontal slices. Note barrels delineated by DAPI staining. (ii) Composite image showing co-expression of DREADD and S100b (yellow). (iii) Image of NeuN staining (blue). (iv) DREADD-mCitrine (green). (v) S100b staining (red). Images below show staining in the expanded region. (vi) The expanded region from (ii). (vii) The expanded region from (iv). (viii) the expanded region from (v). **(C)** Image of an *ex vivo* slice from an animal transfected with GFAP-Melanopsin-CFP and a bulk loaded with Fluo-4AM. Fluorescence traces from circled CFP positive astrocytes are shown below, illustrating calcium elevations in response to light stimulation (blue dashes). **(D)** Example voltage clamp recording from an L2/3 cortical neuron in an *ex vivo* slice from a WT animal transfected with GFAP-melanopsin, illustrating an increase in excitatory current frequency, following 470-nm light stimulation (blue flashes), and example recording from a similar experiment in an IP_3_R2^– /–^ slice. A scale bar, 500 pA, 100 s (expanded inset scale bars, WT 200 pA; 10 ms. IP_3_R2^– /–^ 20 pA, 100 ms). Bar graphs on right show summary effect of 470-nm stimulation in slices from WT (white bars) and IP_3_R2^– /–^ (red bars) animals expressing GFAP-Melanopsin on sEPSCs. **(E)** Data from *in vivo* recordings showing (i) example spike activity recording from a control non-melanopsin animal before and (ii) after 470-nm stimulation. An upper trace depicts raw recorded data with detected spike activity in a below plot. (iii) Similarly, data from a GFAP-melanopsin-expressing animal before and (iv) after 470-nm stimulation. A plot on right (v) Summary data of recordings from control animals (open circles) and animals transfected with GFAP-melanopsin (filled circles) showing a 470-nm light-dependent increase in neuronal firing in the barrel cortex in melanopsin-expressing animals. **(F)** Field recordings in response to standard principal whisker stimulation from an animal-expressing Gq-DREADD, showing an increase in amplitude after CNO injection. (i) Example LFP in response to whisker stimulation in control animals before and (ii) after CNO injection. The upper trace indicates timing of whisker deflection, the lower trace indicates LFP. (iii) Similarly, data from a GFAP-DREADD-expressing animal before and (iv) after CNO injection. (v) Summary data of recordings from control animals (open circles) and animals transfected with GFAP-DREADD (filled circles), showing a CNO-dependent increase in LFP response in the barrel cortex in GFAP-DREADD-expressing animals (0–60 s and 180–240 s shown). CNO injected at time = 0 s.

We expressed either melanopsin or hM3Dq-DREADD in astrocytes using a GFAP promoter (Lee et al., 2008). AAV injections were directed at the mouse barrel cortex. Horizontal slices revealed the pattern and extent of transgene expression. The transgenes were expressed in an oval area with dimensions (length x width): Melanopsin 1,357 ± 92.45 × 785 ± 58 μm, DREADD 1,155 ± 108 × 710 ± 81 μm and covered multiple barrels (Figures 2A,B). Four features of the subsequent immuno-histochemical staining demonstrated that expression of the transgenes was, indeed, confined to astrocytes (1) all melanopsin transfected cells stained for the astrocytic marker S100b (26/26, Figure 2A) (2) none of the melanopsin positive cells stained for the neuronal marker NeuN (0/406, not shown) (3) all DREADD-transfected cells stained for astrocytic markers (30/30, Figure 2B) and (4) none of the DREADD positive cells stained for neuronal markers (0/192, Figure 2B). The data, therefore, confirm selective astrocyte targeting.

In acute cortical slice preparations from WT animals expressing melanopsin in astrocytes, stimulation with light elicited [Ca^2+^]_*I*_ increases in CFP-positive astrocytes (*n* = 3 slices, two mice; Figure 2C). In six out of sevan patch-clamp recordings from L2/3 neurons in melanopsin WT-transfected slices, an increase in the frequency of large amplitude excitatory currents (> mean + 2 SDs) was seen following light stimulation (ctrl: 0.53 ± 0.28; 470 nm; 1.49 ± 0.42; *n* = 7 slices, 5 mice, *p* = 0.034, *t*-test, Figure 2D). In recordings from melanopsin-transfected IP_3_R2^–/–^ slices, there was no light induced increase in large events (ctrl: 1.09 ± 0.29; 470 nm; 1.07 ± 0.29; *n* = 6 slices, 4 mice, *p* = 0.94, *t*-test). The large EPSCs are most likely the result of an increase in neuronal network driving synaptic currents. These results, therefore, indicate that the astrocyte mechanism of increased local neuronal activity is specifically due to activation of IP_3_R2 astrocytic signaling, and that, in the absence of this signaling, Gq activation is ineffective.

To determine whether such astrocyte activation could affect neuronal function *in vivo*, extracellular recordings were made from the barrel cortex of GFAP-melanopsin-transfected mice. We found that light stimulation (470 nm), indeed, caused an increase in spontaneous neuronal activity compared to mice with no light stimulation (Figure 2E) (Mann–Whitney *U* test: *p* < 0.0001, Ctrl: *n* = 13 cells, 3 animals; melanopsin-GFAP: *n* = 32 cells, 11 animals). To determine whether astrocyte Gq-IP_3_ signaling could modulate synaptic efficacy, we then used a chemogenetic approach. When CNO was injected into mice expressing Gq-DREADD exclusively in astrocytes (Figure 2B), during a standard whisker deflection paradigm (Glazewski and Fox, 1996), DREADD activation resulted in an increase in whisker-evoked neuronal responses. We found that L2/3 barrel cortex (LFPs, indicative of population of neuronal responses) increased significantly following CNO injection in DREADD expressing animals compared to sham control animals (Figure 2F) (Baseline, −20 to 0 min Control-CNO: 100.2 ± 2.134; DREADD-CNO: 100 ± 2.734 *p* = 0.983. 0–60 min post injection; Control-CNO: 97.16 ± 1.873, DREADD-CNO: 112.7 ± 1.883, *p* = 0.0000004453. 180–240 min post injection; Control-CNO: 100.7 ± 2.403, DREADD-CNO: 142.3 ± 3.78, *p* < 0.000000000004121, Mann–Whitney *U* test).

These data demonstrate and establish that, in the mouse barrel cortex of adolescent mice, astrocyte Gq-IP_3_-mediated pathways have the capacity to influence local L2/3 neuronal activity and plasticity.

### Neuronal activity is not impaired by IP_3_R2 deletion

We then sought to determine whether or not Gq-IP_3_-mediated pathways in astrocytes had a role in Hebbian and homeostatic EDP. To achieve this, we utilized an IP_3_R2^–/–^ mouse model (Li et al., 2005). The mouse model has been reported to not have noticeable phenotypic or behavioral deficits or features (Petravicz et al., 2008, 2014; Agulhon et al., 2010; Takata et al., 2013; Bonder and McCarthy, 2014; Guerra-Gomes et al., 2020).

**FIGURE 3:**
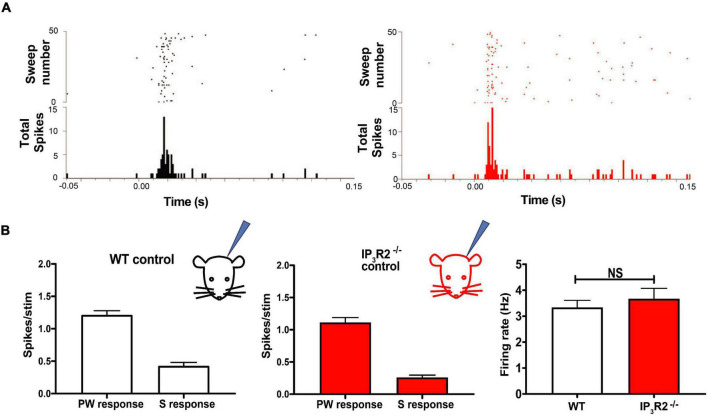
IP_3_R2 mutation neither impairs whisker-evoked neuronal responses nor spontaneous activity recorded in the barrel cortex. **(A)** Raster plots illustrating spiking in barrel cortex in response to a standard principal whisker deflection in WT (black) and in IP_3_R2^– /–^ mice (red). Post-stimulus histograms below show distribution of spikes for the representative trials. **(B)** Bar graphs of quantification of principal (PW) and the surround (S) whisker-evoked responses across all animals in the study for WT (left) and IP_3_R2^– /–^ mice (right), showing similar magnitude of responses. Right most: Bar graphs displaying comparison of spontaneous firing in WT and IP_3_R2^– /–^ animals and the lack of significant difference.

To test whether astrocyte IP_3_R2 receptors affect basal sensory synaptic transmission in the whisker-barrel cortex, we conducted *in vivo* extracellular recordings in IP_3_R2^–/–^ mice. Recordings were made from identified barrel columns of the somatosensory cortex. Responses to whisker deflections were recorded in both WT and IP_3_R2^–/–^ mice (Figure 3A). We found no significant difference in neuronal firing between WT and IP_3_R2^–/–^ mice in their response to stimulating the PW or surround whisker evoking the largest response (S1) (see Figure 1C for definitions) WT: PW- 1.20 ± 0.06, S1: 0.43 ± 0.05, *n* = 9 animals; IP_3_R2: PW- 1.11 ± 0.07, S1-0.26 ± 0.03, *n* = 8 animals, PW: *p* = 0.3665, S1: *p* = 0.2812, Mann–Whitney *U* test; Figure 3B. Similarly, there was no difference in the amount of spontaneous activity recorded in the two groups. WT: 3.34 ± 0.27, IP_3_R2^–/–^: 3.68 ± 0.39, *p* = 0.53, Mann–Whitney *U* test; Figure 3B. These results indicate that the mutation alters astrocyte responses in the barrel cortex while leaving whisker-barrel neuronal responses unaffected.

### Experience-dependent plasticity induced by single-whisker experience is unaffected by astrocyte IP_3_R2 deletion.

**FIGURE 4:**
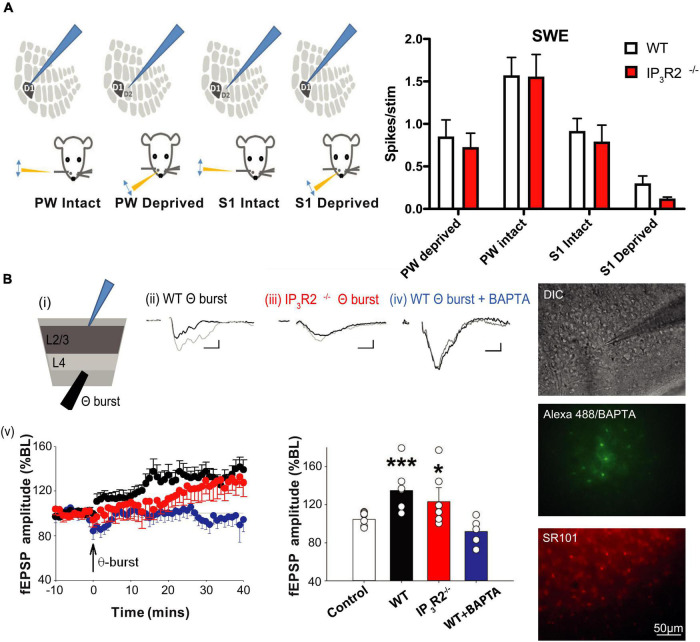
Experience-dependent Hebbian potentiation and *in vitro* long-term potentiation are intact in IP_3_R2^– /–^ L2/3 barrel cortex. **(A)** Experience-dependent Hebbian plasticity induced in the barrel cortex by long-term SWE is unchanged by IP_3_R2 mutation. Illustrations to the left display the four different whisker-deflection barrel cortex response configurations analyzed for the SWE paradigm. The bar graph on the right summarizes data for WT (white bars) and IP_3_R2^– /–^ (red bars) animals. No significant differences between WT animals and the mutants were found comparing PW and S1 responses for all four configurations. **(B)** LTP can be evoked by theta burst stimulation in WT (black) and IP_3_R2^– /–^slices (red). However, introduction of BAPTA into astrocytes blocks LTP induction (Blue). Summary bar graphs of theta burst stimulation experiments under diffferent conditions are shown on the right. Images on the far right show astrocyte BAPTA infusion. Top: DIC image of a slice with an astrocyte patched in L2/3. Middle: The imaged field with astrocytes identified by SR101 loading. Bottom: Including Alexa 488 with BAPTA in a patch pipette enables verification of extent of astrocyte syncytium spread.

With the aim of determining whether IP_3_R2 is necessary for induction of EDHP, we employed the SWE paradigm. All-whiskers apart from one (D1 whisker) were removed from one side of the snout of the animal (Figure 4A) for 18 days, followed by up to a week of regrowth prior to recording. In Layers 2/3 of the adolescent barrel cortex, this protocol is known to result in the potentiation of the intact whisker responses in the immediately adjacent whisker representations and depression of the surrounding deprived whiskers responses in their own representations (Glazewski et al., 1996; Glazewski and Fox, 1996). This scenario was seen in WT animals and in IP_3_R2^–/–^ animals (Figure 4A). Indeed, data from *in vivo* extracellular recordings, there was no significant difference in the magnitude of corresponding PW and SW responses between the WT and IP_3_R2^–/–^-deprived groups (all pairs *p* = 0.125, Wilcoxon-matched pairs signed the rank test, WT: *N* = 11 animals, IP_3_R2^–/–^: *N* = 9, Figure 4A). The data indicate that EDP induced by SWE is not dependent on IP_3_R2 signaling.

### Long-term depression, but not long-term potentiation is dependent on astrocyte IP_3_R2

The capacity for the intact whisker spike response to potentiate following SWE is thought to depend on the presence of LTP at the L4-L2/3 synapse (Hardingham et al., 2003). We, therefore, tested whether LTP was affected in IP_3_R2^–/–^ mice. Single extracellular electrode LFP recordings were performed in L2/3 in acute barrel cortex slices, and theta burst LTP induction protocol was applied in L4 (Figure 4B). We found that LTP was induced in slices from both WT and IP_3_R2^–/–^mice (WT: fEPSP amplitude control, 104.6 ± 4.5%, *n* = 8; LTP, 134.7 ± 9.5%, *n* = 7, *p* = 0.0039, *t*-test; IP_3_R2^–/–^: fEPSP amplitude control, 104.6 ± 2.8%, *n* = 8; LTP, 123.1 ± 14.8%, *n* = 6, *p* = 0.031, *t*-test; Figure 4B), indicating that the IP_3_R2 receptor is not necessary for LTP induction at these synapses.

There is considerable evidence that astrocyte calcium plays a role in hippocampal LTP (Henneberger et al., 2010). Therefore, we patch-clamped an astrocyte in L2/3 with an electrode containing the calcium chelator BAPTA (Figure 4B). By including Alexa488 in the pipette solution, we were able to assess the spatial extent to which BAPTA-buffered calcium in neighboring gap-junction-coupled astrocytes. Control recordings were made where the pipette solution contained Alexa488 without BAPTA. We found that LTP induction was, indeed, blocked when astrocytic calcium was buffered with BAPTA (fEPSP amplitude, 91.9 ± 7.2%; *n* = 5, *p* = 0.0045, *t*-test, Figure 4B). The results indicate that astrocyte calcium signaling is necessary for LTP induction at L4-L2/3 synapses, but that the calcium source may not necessarily be IP_3_R2 dependent.

**FIGURE 5:**
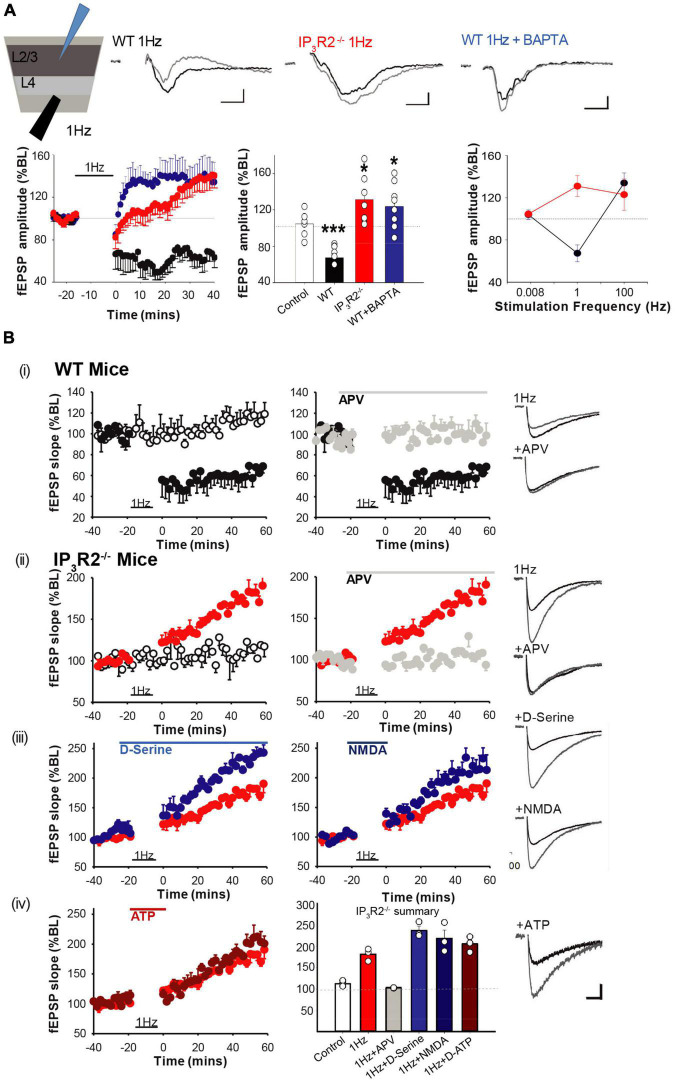
IP_3_R2 is necessary for the expression of *in vitro* NMDAR-dependent long-term depression. **(A)** LTD is evoked by 1-Hz stimulation in WT (black), but not in IP_3_R2^– /–^slices (red). Rather, 1-Hz stimulation results in LTP, which also occurs in WT slices when astrocytes are infused with BAPTA (blue). Significance of changes summarized in bar graphs to the right. A summary plot on the far right of fEPSP amplitude in respons to stimulation frequencies for WT (black symbols) and IP_3_R2^– /–^ (red symbols), displaying leftwards shift in IP_3_R2^– /–^. **(B)** NMDA-dependent LTD cannot be rescued by re-introducing putative gliotransmitters. (i) A plot on the left of the LTD-induction effect (black circles) from MEA recording of 1-Hz LFS on fEPSP responses compared to control stimulation (white circles, once every 120 s). A plot on the right shows that including NMDA antagonist D-APV in the perfusate during 1-Hz LFS blocks induction (gray circles). (ii) In slices from IP_3_R2^– /–^mice, LFS results in potentiation (red circles, a plot on left), which is also blocked by D-APV (right, gray circles). (iii) Plots showing that including NMDAR co-agonist D-serine (blue circles), or the co-agonist NMDA (dark blue circles) in the perfusate, during LFS does not rescue LTD. (iv) Left: Plots showing that including putative gliotransmitter ATP in perfusate during LFS has no effect compared to control. Right: A summary bar graph. Bars display mean ± sem for the experimental group data shown in plots (i)–(iv). Traces to the far right of plots show example fEPSP responses for the experimental conditions at 60 min. A scale bar: 10 ms, 50 μV.

Synaptic long-term depression (LTD) has previously been implicated in EDHD following specific paradigms (Allen et al., 2003; Hardingham et al., 2008); we, therefore, investigated whether LTD induction was affected in IP_3_R2^–/–^ mice. Using single electrode extracellular recordings, a 1-Hz LFS LTD induction protocol was applied to L4 while recording LFP at L2/3 in acute brain slices from the WT and IP_3_R2^–/–^ mice (Figure 5A). It was possible to induce LTD in the slices from the WT mice, but not in the slices from the IP_3_R2^–/–^ mice (WT: fEPSP amplitude, 67.4 ± 8.%, *n* = 7, *p* < 0.0006, *t*-test; IP_3_R2^–/–^: fEPSP amplitude, 131.4 ± 10.8%, *n* = 6, *p* = 0.028, *t*-test; Figure 5A). Indeed, a paradoxical potentiation was observed in the mutant. These data indicate a crucial role for astrocytes in LTD induction in L4-L2/3 of the barrel cortex synapses. A similar response was also seen in L4-L5 synapses (189.8 ± 15.2% at 60 min post 1 Hz-LFS). To confirm and verify an astrocytic locus for the IP_3_R2 deficit, BAPTA and Alexa 488 were infused into a patch-clamped astrocyte. In this case, LTD was, in effect, flipped to LTP (fEPSP amplitude, 123.8 ± 9.7%, *n* = 8, *p* = 0.033, *t*-test, compared to no-stim control, *p* = 0.0001 compared to non-BAPTA 1-Hz stimulation), mimicking the situation seen in the LTD induction paradigm in IP_3_R2^–/–^ mouse slices (Figure 5A), thereby confirming the central role of astrocytic Ca^2+^ in L4-L2/3 LTD.

To gain mechanistic insight into 1-Hz LFS-induced depression and the astrocyte-dependent reversal of sign to potentiation, we conducted pharmacological interventions using multielectrode array recording. In WT slices, LTD was blocked by bath application of the NMDA receptor antagonist APV (100 μM): [LFS, 66.06 ± 3.79%, *n* = 4, vs. control, 114.45 ± 2.81%, *n* = 5, *p* = 0.0025, *t*-test, LFS + APV, 100.6 ± 3.97%, *n* = 3 (Figure 5Bi)]. In IP_3_R2^–/–^ slices, LFS-induced potentiation was also blocked by APV (LFS, 181.7 ± 8.46%, *n* = 3, vs. control, 113.88 ± 3.78%, *n* = 4, *p* = 0.002, *t*-test, Figure 5Bii (LFS + APV, 103.5 ± 0.72%, *n* = 3). Therefore, both effects of LFS (i.e., depression in WT and potentiation in IP_3_R2^–/–^ mice) are mediated by NMDA receptors. This suggests that astrocytic IP_3_R2 has a role in metaplasticity.

Impaired LTD induction by buffering astrocytic calcium and by IP_3_R2 deletion suggests a possible deficit in the calcium-dependent release of a gliotransmitter required for LTD. The finding that LTD is NMDAR mediated implicates NMDAR agonists glutamate or the co-agonist D-serine. However, bath application of D-serine (100 μM) failed to rescue LTD and, indeed, increased potentiation further (1-Hz LFS + 100-μM D-serine, 237.05 ± 10.04%, *n* = 3, vs. LFS, 181.7 ± 8.46%, *n* = 3, *p* = 0.013, *t*-test, Figure 5Biii). Due to previous findings of a bell-shaped dose-response effect of D-serine in LTD (Zhang et al., 2008), a lower D-serine dose (10 μM) was also tested, but this was also unable to rescue LTD (LFS + 10-μM D-serine, 210.94 ± 9.38%, *n* = 3, *p* = 0.13, *t*-test, not shown). We also applied receptor agonist NMDA (10 μM). However, this had no significant effect on LFS-induced potentiation in IP_3_R2^–/–^ mice (1-Hz LFS + NMDA, 218.61 ± 19.31%, *n* = 3, *p* = 0.155. *t*-test) (Figure 5Biii). Finally, the combination of NMDA (10 and 20 μM) and D-serine (100 μM) was also unable to rescue LTD (LFS + 20-μM NMDA + D-serine, 207.06 ± 5.22%, *n* = 3, *p* = 0.15, *t*-test, not shown).

The other potential gliotransmitter candidate is ATP, which has been implicated in plasticity in hippocampus (Koizumi et al., 2003; Serrano et al., 2006) and in neocortex (Rasooli-Nejad et al., 2014). However, 100-μM ATP did not result in a rescue of LTD either (LFS + ATP, 205.48 ± 10.49%, *n* = 3, *p* = 0.152, *t*-test, Figure 5Biv). Taken together, the results indicate that IP_3_R2^–/–^ L4-L2/3 synapses appear only to be able to undergo LTP. The inability to rescue cortical LTD, together with the fact that LFS-induced potentiation is boosted by D-serine and NMDA, suggests that there has been a change in glutamatergic signaling.

### WT and IP_3_R2^–/–^ 1-Hz plasticity is mediated *via* non-ionotropic NMDAR action

**FIGURE 6:**
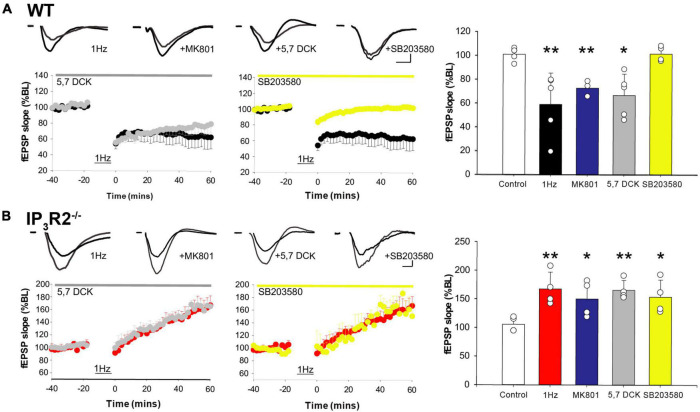
LFS-induced WT long-term depression and IP_3_R2^– /–^ potentiation is mediated by non-ionotropic NMDAR signaling. **(A)** A plot on the left: LTD is induced in WT by 1-Hz stimulation (black) and is not inhibited by a glycine site block (gray), nor an NMDAR channel block (a bar graph, blue). LTD is abrogated by P38 MAPK inhibition (a center plot, yellow). A bar graph on the right summarizes effect of different interventions on WT LTD. **(B)** A plot on the left; LTP is induced in IP_3_R2^– /–^ by 1-Hz stimulation (red) and is not blocked by a glycine site block (gray), nor a channel block (a bar graph, blue). LTP induction is unaffected by P38 MAPK inhibition (a center plot, yellow). A bar graph to the right summarizes effect of different interventions on IP_3_R2^– /–^ LFS-LTP.

A central question that remains is why does a 1-Hz LFS protocol induce potentiation rather than depression when IP_3_R2 astrocyte calcium signaling is abrogated. Historically, it was posited that high levels of calcium induced by High frequency stimulation (HFS) induced LTP, while low levels of calcium induced by LFS induced LTD (Artola and Singer, 1993). However, it is now known that NMDARs can signal non-ionotropically upon glutamate binding and do not necessarily need to flux calcium to induce LTD (Nabavi et al., 2013; Carter and Jahr, 2016). One of the downstream mechanisms that have been proposed is *via* P38 MAP kinase (Aow et al., 2015). We, therefore, investigated this signaling mechanism to address the LTD/LTP dilemma using multielectrode array recording. In WT slices, LFS-induced LTD was not blocked by the glycine/D-Serine site antagonist 5,7 Dichlorokynurenate (DCK) (Figure 6A) (1 Hz: 59.176 ± 11.748% *n* = 6, *p* = 0.0081, *t*-test, 1 Hz + DCK: 55.778 ± 8.04%, *n* = 6, *p* = 0.0035, *t*-test) nor by NMDAR channel blocker MK801 (71.84 ± 5.99%, *n* = 4, *p* = 0.0005, *t*-test), consistent with a non-ionotropic mechanism (Nabavi et al., 2013), but was blocked by treatment with the P38 MAPK inhibitor SB203580 (101.6 ± 3.2, *n* = 4, *p* = 0.9, *t*-test) (Figure 6A). LFS-induced potentiation is also NMDAR-dependent since it is blocked by APV (Figure 5). A possibility to explain the switch from LFS-induced LTD to LTP would be that, in the absence of astrocyte IP_3_R2 signaling, LFS induced an increase in NMDAR-mediated calcium entry, i.e., switched to classical LTP. However, in IP_3_R2^–/–^ slices, LFS-induced potentiation was not blocked by 5.7 DCK (1 Hz: 167.67 ± 14.67%, *n* = 5, *p* = 0.0083, *t*-test, 1 Hz + DCK: 165.78 ± 8.59%, *n* = 5, *p* = 0.0014, *t*-test), nor by the NMDA channel blocker MK801 (150.29 ± 16.04%, *n* = 5, *p* = 0.0006, *t*-test). LFS-induced LTP is, therefore, also independent of ionotropic NMDAR signaling. In contrast to WT LTD, LFS-induced potentiation was not mediated P38 MAPK inhibition (SB203580: 153.86 ± 14.90%, *n* = 5, *p* = 0.025, *t*-test) (Figure 6B), and so non-ionotropic potentiation is not P38 MAPK dependent, and operates *via* a different effector. The loss of astrocyte IP_3_R2 calcium signaling, therefore, leads to a change in postsynaptic signaling, indicating that astrocytes control the synaptic frequency-dependent plasticity response.

### IP_3_R2 is necessary for the induction of experience-dependent depression and triggers homeostatic upregulation

**FIGURE 7:**
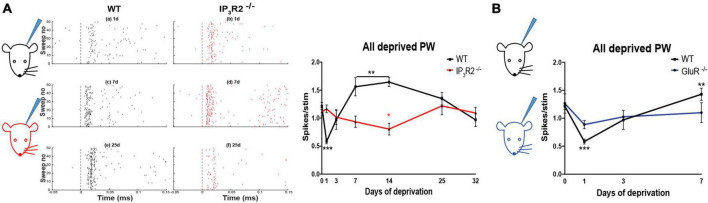
Astrocyte IP_3_R2 is necessary for the induction of experience-dependent Hebbian depression and homeostatic plasticity *in vivo*. **(A)** On the left, an illustration of a unilateral all-whisker deprivation paradigm on mouse snout, with example raster plots of whisker-evoked spike responses in the supragranular barrel cortex at 1, 7, and 25 days post derivation for WT (black) and IP_3_R2^– /–^ mice (red). A plot to the right displays the magnitude of whisker stimulation-evoked spike responses averaged over all recorded animals at times up to 32-day deprivation, showing a stereotypical profile in WT mice (black) involving fast Hebbian depression, followed by slowly developing homeostatic upregulation. Both components are abrogated in IP_3_R2^– /–^ mice (red). **(B)** In GluR^– /–^ mice, there is a reduction in Hebbian depression, following all-whisker deprivation at Day 1 and a reduction in homeostatic rebound at Day 7.

Depriving all the whiskers in WT animals leads to rapid experience-dependent depression, followed by a homeostatic rebound (Glazewski et al., 2017). To test whether astrocyte IP_3_R2 receptors are necessary for the induction of either form of plasticity, we trimmed all-whiskers on one side of the snout of WT and IP_3_R2^–/–^ mice at P28, and made single unit recordings from Layers 2/3 of the contralateral barrel cortex up to 32 days after the deprivation onset. Whisker deprivation was maintained by daily inspection and trimming. WT animals expressed a typical plasticity time course (Glazewski et al., 2017; Figure 7A): at Day 1 post deprivation, there was a marked reduction in the magnitude of response to whisker stimulation in the PW barrel columns. Responses then increased gradually at 3 and 7 days in a homeostatic fashion to overshoot at Day 14 and then reduce to pre-deprivation values by Day 32 (Figure 7A). In contrast, in the IP_3_R2^–/–^ mice, the all-whisker deprivation paradigm caused neither rapid depression, nor a homeostatic rebound, indicating an impairment of the initial depression and subsequent homeostatic upregulation in the IP_3_R2^–/–^ mice. Depression was only detectable after 14 days in IP_3_R2^–/–^animals (1-day depression: *t*-ratio, 7.3, *p* < 0.0001; 7–14-day upregulation: *t*-ratio, 3.7, *p* < 0.003; IP_3_R2^–/–^ decay between P0 and P14: *p* = 0.0426, Mann–Whitney *U* test). Parallel recordings from Layer 4 also indicated an absence of experience-dependent depression and homeostatic upregulation (*F* = 1.525, *p* = 0.21) (data not shown).

### Experience-dependent depression and homeostatic rebound are attenuated in the GluA1 mutant deprived of all-whiskers

An important question that follows from the lack of homeostatic upregulation in IP_3_R2^–/–^ mice is whether IP_3_R2^–/–^ signaling is directly required for homeostasis or whether it indirectly prevents homeostatic potentiation by preventing depression in the first instance.

To distinguish between these possibilities, we made use of GluA1 (GluR1) Knock-out (KO)mice, which are known to not exhibit Hebbian depression in animals that have undergone chessboard whisker deprivation (Wright et al., 2008).

We found that GluA1 KOs exhibited significantly reduced depression following deprivation compared to WTs, and, also, little rebound potentiation. A two-way ANOVA showed an effect of deprivation [*F*_(3_, _3)_ = 9.01, *p* < 0.0005] and an interaction between deprivation and genotype [*F*_(3_, _3)_ = 3.08, *p* < 0.05]. *Post-hoc t*-test showed that this was due to a difference in the degree of depression at 1 day, which was smaller in the GluA1 KOs than in WT [*t*_(8)_ = 3.78, *p* < 0.01] and due to the lack of rebound potentiation seen in the wild types at 7 and 14 days, but not present in the GluA1 knockouts (Figure 7B). The principal whisker responses at 7 days were not different from those in undeprived control GluA1 KOs [*t*_(8)_ = 0.64, *p* = 0.54], indicating no overshoot. Consequently, the response in GluA1 KOs at 7 days was significantly different from that in wild types [*t*_(7)_ = 2.46, *p* < 0.05]. The results are consistent with the hypothesis that homeostatic rebound is triggered by the prior depression, and, therefore, it is sufficient for the IP_3_R2 knockout to impair depression, as it does, in order to prevent homeostatic rebound.

## Discussion

The results of this study show that astrocytes *via* IP_3_R2 signaling play a central role in barrel cortex plasticity, revealing mechanistic roles in Hebbian synaptic plasticity, and, importantly, establishing that astrocytes are necessary for cortical EDP.

Opto- and chemo-genetic stimulation of L2/3 astrocytic Gq-PLC-IP_3_-coupled receptors elicit [Ca^2+^]_*i*_ elevations *in vitro* and result in the increase of barrel cortex neuronal firing *in vitro* and *in vivo*. In conjunction with stimulated whisker input *in vivo*, chemogenetic astrocyte Gq activation results in *de novo* potentiation. In L4 to L2/3 synapses in the WT mice, astrocyte calcium signaling is required for both LTP and LTD induction. In the absence of astrocyte IP_3_R2 signaling, 1-Hz LFS stimulation actually led to a “flip” from LTD to potentiation, suggesting a controlling role for astrocyte calcium signaling in metaplasticity. Both WT LTD and “flipped” LFS-LTP in the mutant rely on a non-ionotropic NMDAR signaling mechanism, but only LTD is P38 MAPK dependent. In contrast to LTD, θ-burst LTP could be induced in the knock-out of IP_3_R2 signaling. *In vitro* findings were reflected *in vivo* so that SWE deprivation still resulted in the potentiation of whisker-evoked responses in the absence of IP_3_R2 signaling, while, with all-whisker deprivation, neither experience dependent depression nor subsequent homeostatic upregulation was induced.

Our data add to and extend the mounting evidence of a central role for astrocytes in cortical function, including plasticity (Navarrete et al., 2012; Perea et al., 2014). In the barrel cortex, astrocytes are necessary intermediaries for the induction of spike timing-dependent depression during early postnatal development (Min and Nevian, 2012), as well as for the cholinergic modulation of cortical Hebbian synaptic plasticity in adolescence (Takata et al., 2011). The astrocyte signaling pathway usually implicated in these roles is the IP_3_-induced release of intracellular calcium, leading to release of gliotransmitters. Optogenetically induced astrocyte calcium elevations have been shown to modulate cortical network state (Poskanzer and Yuste, 2016) and the response selectivity of neurons in the visual cortex (Perea et al., 2014). However, the use of channel rhodopsin (Perea et al., 2014) or archaeorhodopsin (Poskanzer and Yuste, 2016) may not closely mimic physiological astrocyte calcium signaling. In our study, we used a melanopsin optogenetic construct, which, upon light activation, acts *via* Gq to stimulate IP_3_ production (Mederos et al., 2019). Our results show that activation of this central astrocyte physiological pathway leads to increase in spontaneous firing and, importantly, the induction of plasticity to whisker-evoked input, likely by gliotransmitter release (Parri et al., 2001; Fellin et al., 2004; D’Ascenzo et al., 2007; Perea and Araque, 2007). An effect of astrocyte activation on *de novo* potentiation has also been seen in the hippocampus (Adamsky et al., 2018).

### Experience-dependent plasticity

**FIGURE 8:**
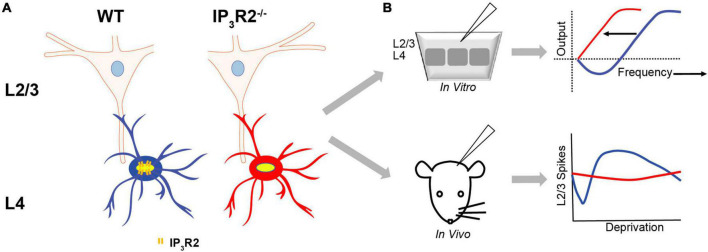
Astrocyte IP_3_R2 signaling in *in vitro* plasticity and experience-dependent plasticity. **(A)** Astrocytes at L4-L2/3 barrel cortex synapses. WT astrocytes (blue) have IP_3_R2 receptors, while IP_3_R2^– /–^ astrocytes (red) are deficient in astrocyte IP_3_R2 receptors. **(B)** An *in vitro* slice (top) and *in vivo* mouse (bottom) recordings reveal that astrocyte IP_3_R2 (WT-blue, IP_3_R2^– /–^-red) controls a synaptic plasticity threshold according to the BCM theory, and the induction of the homeostatic rebound following depression (the bottom right).

The most significant finding in this study is that astrocytes are essential for major forms of EDP. Although EDHD induced by whisker deprivation is a well-known phenomenon (Glazewski and Fox, 1996; Glazewski et al., 1998; Jacob et al., 2007), this present study is the first to investigate the role of astrocytes. Surprisingly, we found that EDHD induced by all-whisker deprivation was impaired in the IP_3_R2^–/–^ mice, while SWE-induced EDHP was not. *In vitro* LTD was also affected (Figure 8).

It is of note that surround deprived whisker responses (deprived whisker stimulated and recording performed in the immediately adjacent-deprived barrel column) are never changed (potentiated or depressed) in a statistically significant manner in the univibrissa-reared animals (Glazewski et al., 1996, 1998; Glazewski and Fox, 1996). The range of variation of the magnitude of the deprived surround input responses that we observed is consistent with that reported in various genetic contexts (for example, Glazewski et al., 1996). Despite the best technical efforts at controlling anesthesia depth, it is possible that observed variations are due to minor depth fluctuations. Lastly, as far as we are aware, surround-deprived responses have never been studied in depth in contrast to principal-deprived responses, both *in vivo* and *in vitro* (Glazewski et al., 1996, 1998; Glazewski and Fox, 1996; Hardingham et al., 2003; Wright et al., 2008).

While synaptic mechanisms for LTD during early development and adolescence differ (Bender et al., 2006; Hardingham et al., 2008; Min and Nevian, 2012), experience-dependent depression induced by whisker deprivation occludes LTD at both ages, suggesting that EDHD and LTD share a common mechanism (Allen et al., 2003; Hardingham et al., 2008). This seems to be a conserved general cortical feature since a similar occlusion occurs in the visual cortex (Heynen and Bear, 2001). Our data showing that IP_3_R2^–/–^ mice have a deficit in LTD and a parallel deficit in EDHD, therefore, indicates that astrocyte IP_3_R2 is an essential component of this common mechanism.

In a similar vein, our results demonstrating that the LTP in L4–L2/3 pathway of the barrel cortex can be induced in IP_3_R2^–/–^ mice but in WT is impaired by the astrocyte infusion of calcium chelator BAPTA is also consistent with recent work showing that astrocyte calcium signaling can be sustained by other IP_3_R subtypes (Sherwood et al., 2017). Other non-IP3R2-mediated mechanisms have also been suggested, including G-protein-mediated increases (Srinivasan et al., 2015), and transmembrane calcium influx into astrocyte processes (Rungta et al., 2016), and mitochondrial calcium release (Agarwal et al., 2017). Since experience-dependent potentiation and LTP likely share an underlying mechanism (Hardingham et al., 2003), this also likely explains why SWE potentiation *in vivo* is unaffected in the mutant.

The IP_3_R2^–/–^, mutation visibly leads to the abrogation of EDHD only if paired with low cortical activity present in all-whisker-deprived animals (where only spontaneous cortical activity is present). In the single whisker-reared animals, the activity in the deprived cortical columns where EDHD is measured, neighbors the intact whisker representation where spontaneous and whisker-evoked activity is present. High activity in this barrel also increases the activity in the deprived columns. In the barrel cortex, EDHD evoked by SWE was found in developing and adolescent but not in adult animals (Glazewski et al., 1996; Fox, 2002), and, at least in adolescence, it depends on cortical activity and the GluA1 subunit of AMPA receptors (Glazewski et al., 1996; Wallace et al., 2001). However, the mechanism underlying all-whisker deprivation-evoked depression has never been delineated at this depth. However, the amount of depression is greater close to the intact whisker representation (Glazewski and Fox, 1996). This coincides well with the differential effect of the mutation in the single vibrissa-reared and all-whisker-deprived animals. In the former, the mutation is paired with greater propensity for depression due to the whisker-evoked activity in the intact barrel, in comparison to less-depression propensity in the latter where all barrel columns receive no whisker-evoked activity.

In light of our findings that all-whisker and univibrissa deprivation results in different outcomes on depression (i.e., abrogated in all-whisker, unchanged in uni-vibrissa), it would be desirable to determine spontaneous cortical activity in the two situations. However, since the EDP is actually induced in a freely moving phase between deprivation and recording days, this question would have to be addressed by recording continually in these awake behaving mice. Such experiments may be possible in future studies. It is, however, known that the firing rate after whisker deprivation lowers the firing rate in immobilized unanesthetized awake rats (Kelly et al., 1999).

### Astrocyte IP_3_R2 controls metaplasticity of low frequency stimulation-induced hebbian plasticity polarity

A standard 1-Hz LFS protocol that induced LTD in WT animals failed to induce LTD in the IP_3_R2^–/–^ animals. Rather, the 1-Hz protocol flipped the change of synaptic gain from LTD to LFS-LTP. This effect was recapitulated in WT slices after acute infusion of astrocytes with BAPTA. The results, therefore, show that LTD expression is dependent on IP_3_R2 signaling, and that the LFS potentiation is astrocyte calcium independent, and, therefore, a separate mechanism to theta stimulation-induced LTP. The results show that the gain change and its direction are differentially regulated by both stimulation frequency and astrocyte calcium signaling. In hippocampus, the block of LTP by calcium clamping can be rescued by addition of D-Serine; however, in this study on the barrel cortex, the application of the co-agonist D-Serine, as well as NMDA, which has been shown to induce LTD itself (Lee et al., 1998), was not able to rescue LTD in IP_3_R2^–/–^ nor was ATP, a Gliotransmitter that can induce synaptic depression (Yamazaki et al., 2003). In addition, rather than rescuing LTD, D-Serine and NMDA addition actually increased the paradoxical 1-Hz-LFS potentiation seen in IP_3_R2^–/–^ slices and following BAPTA infusion. The lack of ability to rescue LTD may indicate that the spatiotemporal activation of extrasynaptic and synaptic NMDARs cannot be mimicked by bath application of putative gliotransmitters, or that synapses are already maximally depressed, and so can only undergo potentiation, a phenomenon known in somatosensory and the visual cortex (Hardingham et al., 2008; Kaneko et al., 2008).

Our finding that WT LTD is dependent on non-ionotropic NMDAR signaling *via* P38 MAPK signaling is consistent with previous studies on hippocampus (Nabavi et al., 2013) and somatosensory cortex (Carter and Jahr, 2016). Our data showing that IP_3_R2^–/–^ LFS-induced potentiation is also mediated by non-ionotropic NMDAR signaling are, however, unexpected, and, as far as we are aware, novel. Our discovery that this flipping of potentiation sign can be achieved by acute astrocyte Ca^2+^ chelation indicates that the mechanism underlying potentiation is extant in L2/3 neurons but, in WT, overridden by astrocyte-signaling LTD. In summary, a loss of astrocyte signaling results in a switch from non-ionotropic depression to non-ionotropic NMDAR potentiation. In the hippocampus, it has been found that LTD induction was dependent specifically on astrocyte P38 MAPK (Navarrete et al., 2019). This could also be the case in the somatosensory cortex; however, in comparison to our results with infusing BAPTA in WT slices, it would be expected that P38 MAPK inhibition block would also lead to LFS-induced potentiation in our preparation. We did not see this; therefore, it is likely that P38MAPK is expressed not only in astrocytes and that its expression in neurons or other cell types has a role in neocortical plasticity. It should also be noted that the hippocampal LTD recorded by Navarrete et al. (2019) was blocked by MK801 and, therefore, is ionotropic NMDAR-dependent. Additionally, hippocampal LFS did not lead to LFS-induced potentiation in the IP_3_R2^–/–^. These differences indicate that the properties of CA1 synapses are different from those in the somatosensory cortex that mediate EDP. The signaling pathway underlying the flipped LTP is unclear at present, but P38 MAPK is not involved. It is known, however, that NMDA signaling non-ionotropically *via* subunit NR1 can act *via* numerous messengers (Rajani et al., 2020). The intracellular pathways mediating the 1-Hz-induced potentiation, therefore, remain to be determined. It is known that astrocyte glutamate release can activate distinct extrasynaptic receptors to those which are activated by synaptic transmission (Fellin et al., 2004; Angulo et al., 2004). Together, therefore, the data on plasticity sign “flipping” could be consistent with a situation where the astrocyte-released GT glutamate in WT activates a separate population of P38 MAPK-linked NMDA receptors that have primacy and mediate LTD, while synaptic glutamate activates a different population, where the NMDAR P38MAPK pathway has dominance. Additionally, the failure to rescue in the IP_3_R2^–/–^ mice may indicate that the separate depressive pathway has not been retained.

### Astrocytic calcium role in experience-dependent homeostatic plasticity

Our data also revealed an impairment of the subsequent homeostatic upregulation, which typically follows a period of greatly decreased activity, indicating a critical role for astrocyte IP_3_R2 in HP. While hippocampal studies have also implicated glia in synaptic-scaling HP (Stellwagen and Malenka, 2006), ours is the first description of a role in experience-dependent homeostatic regulation. However, a further question that arises from our experiments was whether astrocyte IP_3_R2 in HP had a “direct” or “indirect” effect. So is astrocyte IP_3_R2 signaling necessary for both the expression of LTD/EDHD and independently for the induction of homeostatic potentiation (direct), or is the reduction in cortical activity as a result of LTD/EDHD the trigger for homeostatic rebound (indirect)? The small reduction in response after 14 days in the IP_3_R2^–/–^ mouse pointed to the latter scenario so that a prolonged reduction in whisker input does lead to a diminished effect on cortical activity, but does not reach the threshold achieved by the astrocyte-mediated EDHD.

We further tested both hypotheses using the neuronal GluA1 mutant, in which EDHD is impaired in “chessboard”-deprived animals (Wright et al., 2008). Our results showing a reduced homeostatic rebound in concert with reduced EDHD, therefore, support the “indirect” rebound mechanism that first requires reduced activity by EDHD induction. Alternatively, it is possible that GluA1 and, perhaps, other mechanisms involved in EDHD expression are also involved in the homeostatic rebound.

Homeostatic plasticity of gain and of the firing rate is often, although not always tightly linked (Turrigiano, 2017). In this study, we found that, in the barrel cortex, the spontaneous firing rate in L2/3 follows the general pattern of synaptic gain changes. This is not always the case. For example, in L5 of the barrel cortex, homeostatic upregulation of gain and the firing rate is correlated for intrinsically bursting but not for regular spiking neurons (Greenhill et al., 2015; Glazewski et al., 2017).

### Astrocyte IP_3_R2 role in Hebbian plasticity is consistent with the BCM sliding threshold theory

The observed shifts of the thresholds of EDHD and LTD in the IP_3_R2^–/–^ mouse are consistent with predictions from the Bienenstock-Cooper-Munro (BCM) theory (Bienenstock et al., 1982). The theory was developed to explain experimental observations of synaptic properties in visual sensory cortex that were dependent on visual experience, i.e., EDP. For example, following monocular deprivation, the theory predicts that, in response to reduced synaptic input, the threshold for inducing Hebbian synaptic potentiation reduces to a level where less-synaptic input is required to induce synaptic potentiation (Cooper and Bear, 2012). BCM theory, therefore, describes an activity-based homeostatic mechanism. Our findings that astrocyte IP_3_R2 deletion also reduces the threshold, that results in a lack of LTD and EDHD indicate that, in barrel cortex, astrocytes are essential components of this homeostatic control mechanism. Our findings also resonate with those of Huang et al. (2012), who showed that Gq-coupled receptor blockade was shown to promote potentiation and proposed a “vertical- shift” of the BCM curve. Our study suggests that this would be consistent with the critical Gq-IP_3_R receptor pathway being in astrocytes.

Here, we report that astrocyte IP_3_R2 signaling is necessary for EDP, specifically the induction of L2/3 experience-dependent homeostatic depression, LTD, and homeostatic upregulation. This extends our understanding of the important roles of astrocyte IP_3_-mediated signaling in several forms of plasticity *induced in vitro* and novel roles of astrocyte IP_3_R2 in *in vivo* EDP.

Our findings, therefore, indicate that future explanations of EDP should consider the role of astrocytic mechanisms.

## Data availability statement

The raw data supporting the conclusions of this article will be made available by the authors, without undue reservation.

## Ethics statement

The animal study was reviewed and approved by Keele University Bioethics committee, Aston University Bioethics committee. Study performed in accordance with the United Kingdom Animals Scientific Procedures Act of 1986.

## Author contributions

HRP and SG conceived and designed the study. RES designed and conducted the *in vitro* experiments. JBB and SG designed and conducted *in vivo* experiments. NMN and AHB designed and conducted MEA experiments. MK conducted primary cell culture. DAN and EJH designed and produced viral constructs. SIJ and JBB conducted the *in vivo* immunohistochemistry staining. JBB performed the analysis. RES and JBB performed the surgeries and injections of the opto and chemogenetic viruses. JBB, SG, and KF analyzed the *in vivo* data. RES, NN, AHB, and HRP analyzed the *in vitro* data. JBB, RES, SIJ, MK, EJH, DN, KF, HRP, and SG wrote the manuscript. All authors contributed to the article and approved the submitted version.
